# Parental Attitudes to Childhood Overweight: The Multiple Paths through Healthy Eating, Screen Use, and Sleeping Time

**DOI:** 10.3390/ijerph17217885

**Published:** 2020-10-27

**Authors:** Miguel Giménez Garcia-Conde, Longinos Marin, Salvador Ruiz de Maya, Pedro J. Cuestas

**Affiliations:** Department of Marketing, Campus de Espinardo, University of Murcia, 30100 Murcia, Spain; miguelgimenezgarciaconde6@gmail.com (M.G.G.-C.); salvruiz@um.es (S.R.d.M.); pcuestas@um.es (P.J.C.)

**Keywords:** childhood obesity, parental attitudes, feeding, body mass index (BMI)

## Abstract

The main goal of this paper is to examine how parental attitudes toward sleeping, screen use, and feeding their child influence the child’s body mass index (BMI) through the child’s behaviors related to time dedicated to sleep and television, and their healthy eating. Using survey data from 908 parents, results show that parental attitudes have a significant influence on children’s behavior and, more importantly, on their BMI. The three forms of a child’s behavior (sleeping, watching TV, and healthy eating patterns) are affected by what parents do and their attitudes toward these three behaviors at home. Results show how changes in parental attitudes are an interesting target to prevent the child’s overweight.

## 1. Introduction

While over 1.9 billion people are currently overweight or obese [1], more than 41 million of these are children, a segment that has shown increases in obesity in recent decades and at higher rates in middle-income countries [2]. The medical, psychological, and sociological literatures have analyzed factors related to overweight based on parental and child behaviors [3,4]. Parents are very important socialization agents to transfer cultural eating and health habits to their children through their own attitudes and behavior. Recent research has shown how parental eating attitudes influence a child’s dietary patterns [5] or correlate with the parental perception of a child’s obesity [6]. However, little is known about how parental attitudes, not only those related to eating, specifically relate to overweight children and the complex paths this influence takes. 

Regarding the area of health behavior, research has specifically called for studies that explore the mediation of behavioral change outcomes by specific changes in cognition [7]. Moreover, a topic of interesting research focuses on the role of parents in the prevention of overweight by inducing healthy behaviors in their children [8]. Parents not only provide genes that have an influence on a child’s Body Mass Index (BMI), they also manage environments for children that may prevent the development of overweight [9]. A healthy home environment fosters a child’s health and well-being, with engaged and skillful parents who model, value, and encourage sensible eating habits and a physically active lifestyle. By promoting certain values and attitudes, by rewarding or reinforcing specific behaviors, and by serving as role models, parents can have a significant influence on their children being normal weight [10].

Most research addressing overweight children has focused on the influence of parental style feeding practices [11], attitudes toward physical activity [12], rules regarding TV/screen watching [13], and bedtime [14]. However, a simultaneous consideration of these three factors (sleeping, sedentary life, and feeding) can contribute to a better understanding of the home/family antecedents of overweight children. 

Taheri [15] suggests a link between shortage of sleep and overweight in children, adolescents, and adults. Additionally, few interventions have been implemented to increase sleep among children to prevent childhood overweight, according to the Chen et al., [16] meta-analysis. Rules and nightly bedtime routines are important to reach sleep quality (wakefulness after sleep onset and sleep continuity) and quantity for infants and toddlers. As Buxton et al. [17] also state, children generally have better sleeping habits in terms of quantity and quality in the presence of household rules and regular sleep–wake routines. The implementation of this pattern of behavior at home is undoubtedly a parent’s decision, which will be determined by their attitudes.

Conversely, low physical activity, or highly sedentary behaviors (e.g., watching TV or use of screen devices) require low body energy expenditure and, therefore, promote child overweight [18]. The reverse is associated with the time spent on physical activities, negatively related to body fatness in children [19]. Several studies have found strong correlations between the prevalence of overweight and the number of hours that young people spend watching TV [20]. Overall, watching TV and screen time promote a child’s overweight [21] and having a TV/screen in the child’s bedroom is an antecedent of child overweight [22]. Screening is harmful, not only for being a sedentary activity but, also, due to its advertising of obesogenic food [23]. The relationship among physical activity, screen time, and body mass index (BMI) starts as soon as 3–4 years old, with the negative effect of physical activity and the positive one of screen time growing as children age [24]. However, physical activity as well as TV watching and screen use at home are affected by parental attitudes toward healthy habits [23]. Children who lived in a family with tight rules governing TV and screen time, or who never watched TV during dinner, or had only one TV in the household or had no screens in their bedroom, had significantly less TV/screen time than their counterparts [25]. Recent studies have shown clear evidence of the strong link between obesity and childhood media exposure [26].

A third relevant factor, as mentioned before, is related to feeding. While part of the childhood overweight problem may lie in the need to increase body energy consumption through physical activity, taking into account the other side of the energy equation, reducing excessive energy intake is also very important to maintain a healthy weight [19]. Healthy eating is about eating the right amount (according to activity and energy expenditure) of the right things (e.g., vegetables), at the right moment (e.g., breakfast) and at the right speed (i.e., slow eating; [27]). The child’s home diet also depends on the availability and accessibility of healthy and unhealthy foods at home. Foods the child can and cannot find at home and parental eating attitudes will define the healthy character of the family’s home environment. Hammons and Fiese [28], for example, found that sharing three or more family mealtimes per week had multiple healthy eating benefits. As such, family meals constitute an additional determinant for a child’s healthy eating. When the child eats healthy food, he/she will have a healthy weight [6]. The child will eat healthy food if the food environment is rather healthy (in terms of availability and parent modeling) and if family meals are frequent and the child has an opportunity to try all foods.

While it is of interest to simultaneously consider how parental behavior influences sleeping, TV exposure, and feeding to explain a child’s overweight, parental attitudes will provide a better understanding of the previously mentioned effects as they antecede parental behavior. As one of the first and main socialization sources, parents provide a dynamic context for children to learn what attitudes and behavior they can adopt and their consequences [29]. Parental attitudes have a strong influence on children’s attitudes and behavior [30], not only through providing examples but, also, by modifying the environment where children develop activities and attitudes [31]. 

**Figure 1:**
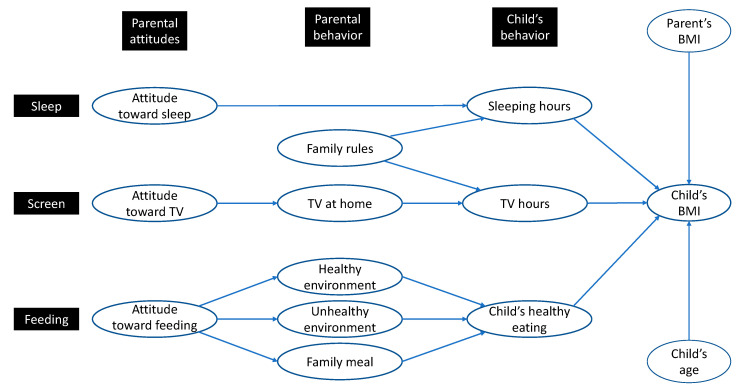
Proposed framework: from parental attitudes to a child’s body mass index.

Based on parental attitudes, parental behavior, and a child’s behavior, we propose a model (Figure 1) with a triple path from parental attitudes to a child’s BMI. The parental attitude–behavior relationship is justified by extensive literature (see [32]), while the relationship between parental and child behaviors is based on considerable evidence, in many cases associated with the term “parenting”, used to explain that relationship [33,34]. 

## 2. Materials and Methods 

### 2.1. Participants and Procedure

We used a cross-sectional survey design to test the proposed framework by contacting the principals of 21 schools in the main cities of the Region of Murcia (Spain) who agreed to participate in the study and distribute the questionnaires. Teachers gave the questionnaire to the children, who took it home and returned it completed by one of their parents. Respondents were 908 parents with at least one child attending primary school in the Region of Murcia, Spain. The person who completed the questionnaire was the parent (mother or father) who usually took care of this child. That person answered questions about the parental attitudes and behaviors toward healthy habits and about the behaviors of the child attending primary school (in terms of eating, TV/screen watching, and sleeping hours). Both the responding parent’s as well as the child’s weight and height were reported to calculate their Body Mass Index (BMI). Data collection took place in the academic year 2014/2015. We clearly informed respondents about the purpose of the study, made clear the anonymous treatment of the data, and obtained their implied consent when they voluntarily agreed to complete the survey.

### 2.2. Measures

Data were self-reported. Parents reported on a child’s behavior by answering questions about sleep, screen time, and eating behavior. A child’s sleeping hours were assessed by asking for the waking-up time and the going-to-bed time. A child’s TV/screen hours were measured by asking the number of hours for which the child watched TV/a screen during weekdays, in 30-min intervals. Concerning a child’s healthy eating, we asked about the number of days per week on which the child consumed fruit, vegetables, and sugared beverages.

A second set of variables measured parental behavior at home. First, we used Adam et al.’s, [35] scale of family rules. To measure if the parents fostered TV/screen watching at home through availability, we asked how many TVs/screens they had at home and if the family watched TV/screen while dining together. The home healthy eating environment was measured with four items related to the availability of healthy foods for children at home (low energy-dense food such as fruit and vegetables) and how often the parents showed model healthy behavior by eating fruit and vegetables. An unhealthy home eating environment was measured with three items about how often parents enabled the child to perform an unhealthy eating behavior (e.g., sugared drinks). We built the healthy/unhealthy scales by taking into account deep interviews with three parents and findings about two relevant aspects: parental modeling and healthy food availability. The items concerning family rules were based on Brown and Ogden’s [36] findings about how a positive parental role model may be a better method for improving a child’s diet than attempts at dietary control (1 = never, 5 = always). The study of Hearn et al.’s, [37] about the relevance of the availability and accessibility of fruit and vegetables was the second source of items for the healthy/unhealthy scales. The last parental behavior included in the questionnaire was related to family meals. We adapted Hendy et al.’s, [38] scale to account for the frequency of dining together and frequency of eating the same food, with no special food for children. 

We adapted Bagozzi and Yi’s [39] research on attitude measurement to account for parental attitudes toward a child’s sleeping and screen time (we measured the degree of agreement from 1=none to 5=very much). Parental attitudes toward feeding their children were built with items from the Child Feeding Questionnaire [40]. Parent and child BMIs were calculated based on the parental reports of theirs and the child’s weight in kilograms and height in meters. We used the International Obesity Task Force (IOTF) BMI cut-offs to define overweight (25–30 kg/m^2^) and obesity (≥30 kg/m^2^).

### 2.3. Statistical Analysis

Descriptive statistics were calculated for sociodemographic characteristics and parent and child weights. We used structural equation model analysis to test the set of relationships described in the proposed framework. The test implied a two-step procedure running, first, a confirmatory factor analysis and a reliability analysis, followed by the estimation of the structural equation model. Statistical analyses were performed using R 3.6.1 [41] with the **psych** (v1.8.12; [42]) and **lavaan** 0.6-4 [43] packages. 

## 3. Results

### 3.1. Sample Description

Mothers outnumbered (83.37%) fathers in the sample, and 39.53% of them had a university degree. The proportion of overweight parents (BMI > 25) was 35.13%, with significant differences between males (55.63%) and females (31.04%; χ^2^ = 33.39; *p* < 0.01). We also found significant differences in the rate of overweight between parents with a university degree (25.63%) and parents with a lower educational level (40.20%; χ^2^ = 19.46; *p* < 0.01). 

The sample of children was almost equally distributed in terms of gender (51.28% girls) and age ranged from around 5 to 14 years old (mean = 9.13). Regarding the children, 28.65% were overweight or obese, with no significant difference between boys and girls (χ^2^ = 0.09; *p* > 0.76). Children of overweight parents had a higher rate of overweight (32.91%) compared to children from normal-weight parents (26.33%; χ^2^ = 4.33; *p* < 0.05). We also found that children had a lower BMI when the parents had a university education (17.54) than when they did not (18.08; t(837) = 2.44; *p* < 0.05).

Most of the children in the sample were the oldest child at home (55.51%), while 33.81% were the second sibling and only 10.68% were in the third or higher position. Just 19.16% of the children ate lunch at school. Only one family said they had no TV at home, 21.37% had only one, 43.06% had two, and 35.46% of the families had three or more TVs/screens at home.

### 3.2. Descriptives, Reliability and Validity

Means and standard deviations for the items measured in the questionnaire and involved in the theoretical model are described in Table 1. 

Four variables were measured through multi-item scales, three related to parental attitudes and one to their behavior. We first calculated Cronbach’s alphas as reliability coefficients for these scales. While the variables of attitude toward sleep and family rules reached acceptable values over 0.70 (0.88 and 0.72, respectively), we had to remove the third item of the variables, attitude toward feeding, to obtain an alpha close to 0.70. Moreover, the alpha for the two items combined to account for the attitude toward TV/screens was 0.24. This low reliability may be due to the combination of regular and reversed items [44] and, as such, we decided to use only the second item in subsequent analyses. 

The multi-item scales were evaluated through confirmatory factor analysis (CFA), using maximum likelihood estimation with robust standard errors and the Satorra–Bentler scaled test. The goodness-of-fit statistics for the model showed satisfactory values: χ^2^ (19) = 66.90, *p* ≈ 0.00, root mean square of approximation (RMSEA) = 0.057, standardized root mean square residual (SRMR) = 0.031, non-normed fit index (NNFI) = 0.944, confirmatory fit index (CFI) = 0.966. Average variance extracted (AVE) and composite reliability (**ρ**) were over the threshold values of 0.50 and 0.60, respectively [44], for attitude toward sleep and family rules. However, while composite reliability was over 0.6 for attitude toward feeding, the AVE was only 0.39. To improve the last measurement, we removed the third item, attitude toward feeding, and applied CFA again. Fit statistics were satisfactory (χ^2^ (17) = 14.49, *p* ≈ 0.21, RMSEA = 0.019, SRMR = 0.020, NNFI = 0.995, CFI = 0.998) and reliability results improved (Table 2).

To assess these multi-item scales’ validity, we checked convergent and discriminant validity. The three constructs showed convergent validity as all the parameters of the indicators were statistically significant (t > 1.96) and greater than 0.50. As a test of discriminant validity, we first checked that none of the confidence intervals of the correlations among the latent constructs (Φ-matrix, see Table 3) included the value of 1 [39]. Additionally, we tested that the AVE exceeded the square of its correlation with the rest of the latent variables for each latent variable.

The remaining behavioral variables measured with multiple items shown in Table 1, accounting for days of a particular behavior, were transformed into single measures. We calculated the average of the items included in each variable. Concerning the variable healthy eating, we reversed the third item that then accounted for the days the child did not drink sugared soda. Regarding family meals, we also reversed the second item, then accounting for the days the parent does not prepare a dinner especially for the child.

The variables included in our theoretical model, as shown in Figure 1, were measured as follows: three multi-item scales (attitude toward sleep, attitude toward feeding, and family rules) and nine single-item variables (attitude toward TV/screen, availability of TV at home, healthy environment, unhealthy environment, family meal, sleeping hours, TV/screen hours, healthy eating, and the child’s Body Mass Index (BMI). Shown in Figure 1, in addition to the main core of relationships among parental and child variables, we included the child’s age and the interviewed parent’s BMI in the model as control variables. 

Potential method bias also was evaluated, based on the marker variable technique [45]. Nonetheless, as many correlations in the correlation matrix were smaller than 0.001, the significance of the adjusted correlations did not differ from those of the non-adjusted, which indicates evidence that common method bias was not present in the data. 

Moreover, we took the following precautions to address the possibility of common method bias. First, predictor and criterion variables were distanced as much as possible in the survey instrument by other instrument items not included in this study. Second, protection of respondent anonymity was asserted, as was the fact that there was no right or wrong answer. Third, scale items were constructed by carefully adapting, where possible, extant items from sources that have established reliability and validity. Additionally, items were refined through information obtained from subject interviews and through pilot testing.

### 3.3. Model Estimation

We used structural equation modeling and a robust maximum likelihood estimation with robust standard errors to test the proposed theoretical model. Results showed that the model acceptably fit the data, as evidenced by the goodness-of-fit measures (χ^2^ (132) = 437.728 (*p* = 0.000), RMSEA = 0.052, NNFI = 0.850, CFI = 0.884), and took into account the high number of variables used in the model and the fact that we used a big sample ([44], p. 589). After this first estimation, we removed the path from a child’s healthy eating to a child’s BMI and added a path from availability of TV at home to a child’s healthy eating based on the reference to dinner. The second estimation also showed acceptable fit (χ^2^ (131) = 433.752 (*p* = 0.000), RMSEA = 0.052, NNFI = 0.850, CFI = 0.885) and a significant reduction in chi-squared (χ^2^ (1) = −3.976, *p* < 0.05).

The results displayed in Table 4 allowed us to prove that parental attitudes influence parental behaviors and that the latter have a significant influence on a child’s behavior and onward to the child’s BMI. Regarding the sleep path, we saw that a child’s sleeping time increased with positive parental attitudes toward sleep (b = 0.07, t = 1.98) and family rules (b = 0.20, t = 4.89). 

Parental attitudes toward TV (screen path) conditioned their behavior, as their attitudes toward TV positively influenced the availability of TV at home (b = 0.16, t = 4.99). However, if we turn to a child’s behavior, we see that the time they spent watching TV increased with the availability of TV at home (b = 0.21, t = 6.49), while this effect was only partially compensated by the negative effect of family rules (b = −0.10, t = 2.68). 

The feeding path also showed significant results, with attitudes having a significant effect on behavior again. Parental attitudes toward feeding had a direct and positive effect on the healthy eating environment at home (b = 0.24, t = 5.23) and family meals (b = 0.26, t = 5.98), while they showed a negative influence on an unhealthy eating environment (b = −0.20, t = 4.25). Interestingly, what parents do concerning food affects what children eat too. The child shows a healthier eating behavior in the healthier the home environment (b = 0.44, t = 15.99) and in the higher the frequency of family meals (b = 0.16, t = 5.13). However, two variables related to parental behaviors had a negative influence on a child’s healthy eating: the initially proposed unhealthy environment (b = −0.15, t = 4.80) and the availability of TV at home (b = −0.06, t = −2.02).

Out of the three variables accounting for a child’s behavior, only two of them significantly affected a child’s BMI. While more sleeping hours reduced a child’s BMI (b = −0.08, t = 2.42), the number of hours they spent watching TV per day positively contributed to their BMI (b = 0.13, t = 4.50). These effects were significant, even controlling, for the effects of a parent’s BMI (b = 0.26, t = 8.14) and a child’s age (b = 0.17, t = 4.11). The third proposed antecedent, healthy eating, was not significant in the first model and was removed in model 2. This lack of effect was not due to the presence of the other antecedents or the control variables in that regression, but to the lack of correlation between the two variables (−0.005). The model explained a low proportion of the variance of a child’s BMI (R2 = 0.12) and a higher proportion of their healthy eating (R2 = 0.26). To summarize, all of the proposed relationships in the conceptual model, except one, were supported, with two unrelated dependent variables. 

## 4. Discussion

Families provide the first socialization environment to children, with parents being the core socializing agents. A healthy household environment will be a source of information and good practices for children learning the culture in which they live. Actually, children perceive parents as the most effective source in encouraging them to eat healthy food [46].

However, while literature has shown correlations between parental behaviors and a child’s weight, our research considers an additional stage to better understand parental behavior: parental attitudes. As attitudes predict behavior, and changing them may be easier [47], our results first contribute to the literature on a child’s obesity by demonstrating how changes in parental attitudes are an interesting target to prevent a child’s overweight. 

The combination of the three groups of antecedents (parental attitudes and behavior and child’s behavior) and the three paths allowed us to confirm a significant influence of parental attitudes and behaviors on their child’s Body Mass Index (BMI)and healthy eating habits. Moreover, the influence on BMI also is mediated by a child’s behaviors related to sleeping and TV/screen viewing. These significant relationships are the core of our second contribution, since our findings suggest that parental consistency between what they like (attitudes) and what they do (behavior) significantly influences their child’s healthy behaviors. As such, parents could capitalize on this opportunity to role model healthful behaviors that will contribute to preventing their children being overweight.

Our results are consistent with previous findings about the negative relationship between sleep duration and risk of a child’s overweight or obesity [16], while we contribute to a better understanding of the parents’ role in a child’s sleeping habits. Our model demonstrates that the family rules parents implement, and their attitude toward the relevance of sleep condition, affects the time for which their children sleep. A similar reasoning can explain the contribution in terms of the time children dedicate to TV and screens. Parents have a significant role in this childhood behavior too. Moreover, our findings also are consistent with previous literature on nutrition and psychology [48] that has demonstrated the direct influence of parental behavior on a child’s healthy eating. As De Bourdeaudhuij et al. [49] point out, children who perceive a social environment supportive of fruit and vegetable intake also report the availability of fruit and vegetables at home.

Contrary to our expectation, the relation between the child’s healthy eating behavior and his/her BMI is not significant. A possible explanation may rely on the fact that while the child’s BMI is an observed variable, the measurement of the child’s behavior derives from a parent’s estimation of the days the child has an eating behavior fully or partly based on fruit and/or vegetables but no sugared sodas. The parent’s perception may be biased or based on different proportions of fruit and vegetables. Moreover, their perception also may be affected by what they would like to be the case. 

Added to our contribution to the literature, these research findings may be of interest to parents’ associations and public institutions worried about a child’s overweight. Parents are permanent examples for a child’s behavior. As long as parents change their attitudes and behavior, they can contribute to preventing a child’s overweight. Public and health institutions can target parents to make them aware of their relevance in this issue. Their actions may complement those addressed to children as a way to reinforce and favor a child’s behavioral changes associated with sleep, screen time, and eating. Implications also may extend to both health policy-making and clinical practice, such as those related to sleep time. Our results are consistent with a recent meta-review on the relationship between a child’s sleep and health [46] and should be of high interest for pediatricians and educators to analyze the antecedents of a child’s poor school performance. 

While the present research focused on the antecedents of a child’s overweight, future research could examine in which part of the process (i.e., parental attitudes, parental behavior, or a child’s behavior) interventions could be more effective to reduce a child’s overweight. Moreover, additional variables may be included in the model to explain a child’s overweight, such as a child’s physical activity, their attitudes toward healthy behaviors, or the family use of mobile devices. The need for these new variables is supported by the low R-square of a child’s BMI in our model [50]. Nonetheless, while a better explanation of a child’s BMI is needed, we have to acknowledge that the purpose of our study was not to list all of the antecedents of a child’s overweight, but to identify significant ones and the role of parental attitudes. 

Our study, however, has some limitations. First, the study was conducted in a particular region of Spain and schools were not randomly selected, which limits the extrapolation for the entire Spanish population. As this is a cross-sectional study, we cannot rule out effects such as reverse causality. Questionnaires were self-reported, and answers may be affected by a lack of accurate information for variables such as weight or height, or even some social desirability bias, especially for those variables that account for parental and child behaviors.

## 5. Conclusions

Parents are key socialization agents and, as such, permanent examples for a child’s behavior. Their attitudes and behaviors concerning health related activities can show their children ways to prevent overweight. However, research is needed to understand how those parental attitudes and behavior influence children. This paper contributes to the literature by examining how parental attitudes toward sleeping, screen use, and feeding their child influence a child’s body mass index (BMI). Results show those effects are mediated by parent and child behaviors. Parental behaviors consistent with their attitudes show a significant influence in their child’s healthy behaviors. The three forms of a child’s behavior (sleeping, watching TV, and healthy eating patterns) are affected by what parents do, while only the time dedicated to sleep (positively) and watching TV (negatively) significantly contribute to prevent the child’s overweight.

## Figures and Tables

**Table 1:** Means and standard deviations.

Variables and Items			Mean	Standard Deviation
Parental Attitudes				
	Attitude toward sleep (Likert 1–5)			
		Is healthy for my child to sleep a lot	3.53	1.22
		I like my child to sleep for a long time	3.25	1.19
	Attitude toward TV/screen (Likert 1–5)			
		It is harmful that my child watches TV/screen (reversed)	1.91	1.02
		Watching TV/screen is healthy entertainment for my child	1.98	0.90
	Attitude toward feeding (Likert 1–5)			
		I like to be responsible in feeding my child	4.61	0.72
		I like to decide if my child has taken the right foods	4.55	0.71
		I like to decide the size of the portions my child takes	2.56	1.25
Parental Behavior				
	Family rules (1 = never, 5 = always)			
		You require your child to keep his room clean and tidy	4.43	0.81
		You require your child put aside dirty clothes	4.56	0.80
		You set certain limits on TV/screen watching and time to be home	4.24	0.95
		You enforce the rules you establish	4.28	0.80
	Availability of TV at home			
		Days your child has dinner while watching TV/screen	4.13	2.78
		Number of TVs/screens you have at home	2.28	1.01
	Healthy environment (0–7 days in the week)			
		Days you have fruit available at home	6.43	1.27
		Days you serve peeled fruit for dinner	3.36	2.55
		Days you eat fruit	5.40	1.96
		Days you eat vegetables	5.38	1.73
	Unhealthy environment (0–7 days in the week)			
		Days you drink sugared sodas	2.23	2.28
		Days you eat candy, chocolate, or sweets	1.81	1.65
		Days you eat salty snacks: chips, peanuts, etc.	1.72	1.47
	Family meal (0–7 days in the week)			
		Days you have dinner with your child	5.01	2.47
		Days you prepare your child a dinner specially for him/her (reversed)	6.10	1.70
		Days you serve your child a little of each of the meals on the table	4.68	2.60
Child’s Behavior				
	Sleeping hours			
		Number of hours per day that the child sleeps	9.70	0.61
	TV/screen hours			
		Number of hours per day the child watches TV/screen	2.02	1.03
	Child’s healthy eating (0–7 days)			
		Days a week your child eats fruit at home	5.01	2.04
		Days a week your child eats dinner with vegetables	3.26	2.10
		Days a week your child drinks sugared soda (reversed)	5.71	1.67
Dependent Variable				
	Child’s BMI		17.89	3.18
Control Variables				
	Parent’s BMI		24.38	4.16
	Child’s age		8.93	1.75
Gender Distribution				
	Parents (% female)		83.37	
	Children (% female)		51.28	

**Table 2:** Multi-item scales and reliability.

Variables/Constructs and Items	λ ^1^	ρ	AVE	Cronbach’s Alpha
Attitude toward sleep (Likert 1–5)		0.88	0.79	0.88
Is healthy for my child to sleep a lot	0.94			
I like my child to sleep for a long time	0.84			
Attitude toward feeding (Likert 1–5)		0.69	0.53	0.69
I like to be responsible in feeding my child	0.73			
I like to decide if my child has taken the right foods	0.72			
Family rules (Likert 1–5)		0.69	0.43	0.68
You require your child to keep his room clean and tidy	0.76			
You require your child put aside dirty clothes	0.63			
You enforce the rules you establish	0.54			

^1^ Standardized factor loadings.

**Table 3:** Correlation matrix of multi-item constructs.

Constructs	Attitude toward Sleep	Attitude toward Feeding	Family Rules
Attitude toward sleep	1		
Attitude toward feeding	0.04	1	
Family rules	−0.10	0.35	1

**Table 4:** Structural equation modeling results for hypothesis testing. Standardized betas (t-values).

Path	Model 1	Model 2
Parental Attitude toward TV → Availability of TV at home	0.16 (4.99) ***	0.16 (4.99) ***
Parental Attitude toward feeding → Healthy environment	0.24 (5.23) ***	0.24 (5.23) ***
Parental Attitude toward feeding → Unhealthy environment	−0.20 (−4.25) ***	−0.20 (−4.25) ***
Parental Attitude toward feeding → Family meal	0.26 (5.98) ***	0.26 (5.98) ***
Parental Attitude toward sleep → Child’s Sleeping hours	0.07 (1.98) *	0.07 (1.98) *
Family rules → Child´s Sleeping hours	0.20 (4.89) ***	0.20 (4.89) ***
Family rules → Child´s TV hours	−0.10 (−2.68) **	−0.10 (−2.68) **
Availability of TV at home → Child´s TV hours	0.21 (6.49) ***	0.21 (6.49) ***
Availability of TV at home → Child´s Healthy eating		−0.06 (−2.02) *
Healthy environment → Child´s Healthy eating	0.44 (16.04) ***	0.44 (15.99) ***
Unhealthy environment → Child´s Healthy eating	−0.15 (−5.05) ***	−0.15 (−4.80) ***
Family meal → Child´s Healthy eating	0.16 (5.26) ***	0.16 (5.13) ***
Child´s Sleeping hours → Child´s BMI	−0.08 (−2.46) *	−0.08 (−2.46) *
Child´s TV hours → Child´s BMI	0.13 (4.53) ***	0.13 (4.50) ***
Child´s Healthy eating → Child’s BMI	0.00 (0.14) ^ns^	
Child’s age → Child’s BMI	0.17 (4.12) ***	0.17 (4.11) ***
Parent’s BMI → Child’s BMI	0.26 (8.14) ***	0.26 (8.14) ***

* *p* < 0.1, ** *p* < 0.05, *** *p* < 0.01, *ns* = not significant.
